# Blockade of Macrophage CD147 Protects Against Foam Cell Formation in Atherosclerosis

**DOI:** 10.3389/fcell.2020.609090

**Published:** 2021-01-08

**Authors:** Jian-Jun Lv, Hao Wang, Hong-Yong Cui, Ze-Kun Liu, Ren-Yu Zhang, Meng Lu, Can Li, Yu-Le Yong, Man Liu, Hai Zhang, Tian-Jiao Zhang, Kun Zhang, Gang Li, Gang Nan, Cong Zhang, Shuang-Ping Guo, Ling Wang, Zhi-Nan Chen, Huijie Bian

**Affiliations:** ^1^Department of Cell Biology, National Translational Science Center for Molecular Medicine, Fourth Military Medical University, Xi’an, China; ^2^School of Science, College of Life Science and Bioengineering, Beijing Jiaotong University, Beijing, China; ^3^Institutes of Biomedicine and Department of Cell Biology, Jinan University, Guangzhou, China; ^4^Department of Pathology, Xijing Hospital, Fourth Military Medical University, Xi’an, China; ^5^College of Military Preventive Medicine, Fourth Military Medical University, Xi’an, China

**Keywords:** atherosclerosis, foam cell formation, macrophage, CD147, CD36

## Abstract

The persistence of macrophage-derived foam cells in the artery wall fuels atherosclerosis development. However, the mechanism of foam cell formation regulation remains elusive. We are committed to determining the role that CD147 might play in macrophage foam cell formation during atherosclerosis. In this study, we found that CD147 expression was primarily increased in mouse and human atherosclerotic lesions that were rich in macrophages and could be upregulated by ox-LDL. High-throughput compound screening indicated that ox-LDL-induced CD147 upregulation in macrophages was achieved through PI3K/Akt/mTOR signaling. Genetic deletion of macrophage *CD147* protected against foam cell formation by impeding cholesterol uptake, probably through the scavenger receptor CD36. The opposite effect was observed in primary macrophages isolated from macrophage-specific *CD147*-overexpressing mice. Moreover, bioinformatics results indicated that CD147 suppression might exert an atheroprotective effect via various processes, such as cholesterol biosynthetic and metabolic processes, LDL and plasma lipoprotein clearance, and decreased platelet aggregation and collagen degradation. Our findings identify CD147 as a potential target for prevention and treatment of atherosclerosis in the future.

## Introduction

Cardiovascular events, such as myocardial infarction and stroke, which are the leading causes of morbidity and mortality in the world, are most commonly caused by atherothrombotic occlusion of blood vessels (Grebe et al., 2018). Preceding longtime atherosclerotic changes of the blood vessels arise from dyslipidemia and vascular inflammation driven by accumulation of cholesterol-laden macrophages in the artery wall (Moore et al., 2013). Atherosclerosis, the main contributor of cardiovascular events, is initiated by passage of cholesterol-carrying oxidative-low-density lipoprotein (ox-LDL) into the impaired arterial wall. Ox-LDL retention elicits local inflammation, with influx of monocytes that differentiate into macrophages and subsequently take up ox-LDL to form foam cells, which is a central hallmark of atherosclerosis. Increasing evidence suggests that native LDL and acetylated-LDL (ac-LDL) are also taken up by macrophages and contribute to foam cell formation (Liao et al., 2009; Patel et al., 2015). Accumulating studies have shown that the scavenger receptors CD36 and scavenger receptor-A (SR-A) account for up to 90% of cholesterol uptake, resulting in lipid accumulation and foam cell formation (Frodermann and Nahrendorf, 2018; Wang et al., 2019). Foam cells can further trigger a series of inflammatory responses, thereby accelerating plaque progression (Li and Glass, 2002). Notably, as an inflammatory disease, inflammation in atherosclerosis cannot be easily resolved because cholesterol-laden macrophages tend to be immotile and trapped in the plaques in the course of hyperlipidemia (Moore et al., 2013; Frodermann and Nahrendorf, 2018). Therefore, inhibiting foam cell formation is a fundamental step to alleviate atherosclerosis. Despite decades of research, the underlying mechanisms of foam cell formation regulation remains incompletely understood.

CD147 is a member of the immunoglobulin superfamily and a highly glycosylated transmembrane protein, which has a single deglycosylated core protein of 27 kDa. However, its low and high glycosylated mature forms have a molecular weight of 33 kDa and between 43 and 66 kDa, respectively, where the glycosylation varies depending on cell and tissue type (Yu et al., 2006). CD147 was originally found to induce fibroblast secretion of matrix metalloproteinases (MMPs) in tumor cells. In cardiovascular pathologies, CD147 can induce MMP-9 in monocytes and MMP-2 in smooth muscle cells (Braundmeier et al., 2006). CD147 also serves as a novel receptor on platelets that activates platelets and aggravates inflammation in monocytes (Schmidt et al., 2008). Several studies have shown that CD147 is strongly involved in the development of various inflammatory diseases, such as COVID-19 (Wang et al., 2020), rheumatoid arthritis (Guo et al., 2019), and inflammatory bowel disease (Xu et al., 2020). Our previous studies have shown that CD147 participates in reprogramming of glucose metabolism (Huang et al., 2014) as well as in lipid metabolism (Li J. et al., 2015) in hepatocellular carcinoma, including *de novo* lipogenesis and fatty acid-oxidation. Given its function in inflammation and metabolism, we have been committing to determining the role that CD147 might play in atherosclerosis, especially in foam cell formation.

In the present study, we found that CD147 expression is specifically increased in mouse and human atherosclerotic lesions that are rich in macrophages. We demonstrated that CD147 is upregulated by ox-LDL in macrophages through PI3K/Akt/mTOR signaling. We first found that CD147 plays an important role in foam cell formation. Macrophage-specific *CD147* knockout inhibits foam cell formation, whereas macrophage-restricted *CD147* overexpression promotes this process. The underlying mechanism might include altered ox-LDL uptake through regulation of the scavenger receptor CD36. Moreover, our findings indicate that macrophage-specific *CD147* deficiency may protect against atherosclerosis in versatile aspects. Altogether, CD147 may become a potential target for prevention and treatment of atherosclerosis in the future.

## Materials and Methods

### Antibodies and Reagents

Anti-human CD147, FITC anti-human CD147 (53027, Thermo Fisher Scientific), and anti-human tubulin antibodies were produced by our lab (Chen, 1992; Cui et al., 2018; Lu et al., 2018; Wang et al., 2020). The other antibodies used in this study were as follows: Rabbit anti-mouse CD147 (ab188190), anti-human CD68 (ab955), anti-α-SMA (ab7817), anti-ABCG1 (ab52617), and anti-SR-A (ab151707) antibodies were purchased from Abcam (Cambridge, United Kingdom); anti-mouse CD68 (MCA1957) and anti-F4/80 (MCA497) antibodies were purchased from Bio-Rad (California, United States). PE anti-mouse CD147 (562676) antibody was purchased from BD Biosciences (Franklin Lakes, NJ, United States); anti-p-PI3K (4228), anti-PI3K (4292), anti-p-Akt (4058), anti-Akt (9272), anti-p-mTOR (5536), anti-mTOR (2983), and anti-p-p65 (3033) antibodies were purchased from Cell Signaling Technology (MA, United States); PerCP anti-CD11b (101230) and FITC anti-F4/80 (123107) antibodies were purchased from BioLegend (SanDiego, United States); anti-mouse tubulin (EM0103) antibody was purchased from HuaBio (Hangzhou, China); anti-ABCA1 (NB400-105) antibody was purchased from Novus Biologicals (United States); goat anti-mouse CD147 (AF772), anti-CD31 (AF3628), anti-LDLR (AF2255), and anti-CD36 (AF2519) antibodies were purchased from R&D (Abingdon, United Kingdom); anti-IκB (10268-1-AP) and anti-p65 (10745-1-AP) antibodies were purchased from Proteintech (IL, United States); isotype-matched control antibody mIgG was purchased from Sigma-Aldrich (Darmstadt, Germany); horseradish peroxidase-conjugated anti-mouse, anti-rabbit, and anti-goat secondary antibodies and fluorescent secondary antibodies were purchased from Invitrogen (Carlsbad, CA, United States). Ox-LDL, LDL, ac-LDL, DiI-ox-LDL, and HDL were obtained from Peking Union-Biology (Beijing, China). The inhibitor library was purchased from Selleck (Houston, Texas, United States). PMA, Oil Red O, and ApoAI were purchased from Sigma-Aldrich. Bodipy 493/503 (D3922) was purchased from Invitrogen (Carlsbad, CA, United States).

### Mice

C57BL/6J mice were obtained from Vitalstar Biotechnology (Beijing, China), and *Lyz2*^cre/cre^ and *ApoE*^–/–^ mice were obtained from Nanjing Biomedical Research Institute of Nanjing University. *CD147*^f/+^ mice, with two Loxp sites flanking exons 2 and 7 of the *CD147* gene, were constructed in our lab (Yao et al., 2013). To generate macrophage-specific *CD147* knockout (*Lyz2*^cre/+^*CD147*^f/f^, CD147^M–KO^) mice, *Lyz2*^+/+^*CD147*^f/+^ mice were first crossed with *Lyz2*^cre/cre^*CD147*^+/+^ mice. The F1 *Lyz2*^cre/+^*CD147*^f/+^ genotype was further crossed with *Lyz2*^+/+^*CD147*^f/f^ mice to generate *Lyz2*^cre/+^*CD147*^f/f^ mice, which were named CD147^M–KO^ mice. *Lyz2*^+/+^*CD147*^f/f^ mice were used as controls and are referred to as CD147^WT^ mice. To construct macrophage-specific *CD147* knockin mice, we first generated mice heterozygous for floxed STOP CD147 (the *CD147* gene was preceded by a stop codon that was flanked by two Loxp sites) after the *ROSA26* promoter (CD147KI^f/+^) (Cyagen Biosciences, China). To generate macrophage-specific *CD147* knockin (*Lyz2*^cre/+^*CD147*KI^f/f^, CD147^M–KI^) mice, *Lyz2*^+/+^*CD147*KI^f/+^ mice were first crossed with *Lyz2*^cre/cre^*CD147*^+/+^ mice. The F1 *Lyz2*^cre/+^*CD147*KI^f/+^ genotype was self-crossed to generate *Lyz2*^cre/+^*CD147*KI^f/f^, which were named CD147^M–KI^ mice. *Lyz2*^+/+^*CD147*KI^f/f^ mice were used as controls and here are referred to as CD147^WT^ mice. CD147^M–KO^ and CD147^M–KI^ mice were crossed with their own CD147^WT^ mice to reproduce. All genotypes were confirmed by PCR analysis. *CD147* deletion and overexpression in macrophages were confirmed by western blotting and real-time PCR (RT-PCR).

For atherosclerosis model induction, 8 week-old *ApoE*^–/–^ mice were fed a high fat Western diet (TD.88137, Harlan Teklad) for at least 16 w. The same old C57BL/6J and *ApoE*^–/–^ mice that were fed a normal chow diet for the same time were used as controls. Mice were housed in cages under specific-pathogen-free conditions at 22–25°C with 12 h:12 h light-dark cycle in the Fourth Military Medical University.

### Analysis of Lipid Level Profile

Blood was removed from the eyeball of the mouse after an overnight fast and then anesthesia with pentobarbital sodium. Blood was allowed to clot for 30 min at room temperature followed by centrifugation at 4,000 rpm at 4°C for 12 min. Serum was transferred to a new tube and stored at −80°C. Total cholesterol, total triglycerides, HDL-C, and LDL-C were determined by biochemical automatic analyzer (Hitachi7600). The concentrations of ox-LDL in serum from chow-fed C57BL/6J and *ApoE*^–^*^/^*^–^ mice and Western diet-fed *ApoE*^–^*^/^*^–^ mice were measured by ELISA according to the manufacturer’s instructions (Mlbio).

### Immunohistochemical and Immunofluorescence Staining

Multiple parts of human artery tissue and atherosclerosis tissue arrays (AR301, Alenabio) were used for immunohistochemical and immunofluorescence staining. Mouse aortic sinus and aortic arches were embedded in optimum cutting temperature compound and paraffin, respectively and were examined in 5 μm-thick sections. Atherosclerotic plaque morphology was determined after staining with hematoxylin and eosin (H&E). Immunohistochemistry was performed using anti-CD147 antibody and a streptavidin-peroxidase staining kit (Zhongshan Jinqiao). The immunohistochemistry staining was independently assessed by two experienced pathologists. The staining intensity was scored as 0 (no staining), 1 (weakly positive), 2 (moderately positive), 3 (strongly positive). The positive area of staining was graded as 0 (≤5%), 1 (5–30%), 2 (30–70%), and 3 (>70%). The immunohistochemistry score was calculated by staining intensity + positive area score. For immunofluorescence, sections or cells were stained with specific antibodies, followed by fluorescence-labeled secondary antibodies. Cell nuclei were counterstained with 4’,6-diamidino-2-phenylindole (DAPI). The sections were visualized using a fluorescence microscope (Olympus, Tokyo, Japan) or an LCS-SP8-STED confocal microscope (Leica, Germany). The quantification was performed with Image Pro Plus 6 Software.

### Isolation and Culture of Macrophages and Induction of Foam Cells

The human monocytic cell line THP-1 was acquired from the American Type Culture Collection (ATCC, Manassas, VA, United States). To induce foam cells, THP-1 cells were treated with 100 ng/mL PMA for 48 h and then with 50 μg/mL ox-LDL for 24 h. Mouse primary bone marrow-derived macrophages (BMDMs) were collected from the long bones and cultured with DMEM [Supplementary with 10% FBS and 20% L929 (ATCC)] conditioned medium for 5–7 days and incubated with 50 μg/mL ox-LDL for 24 h. Mouse primary peritoneal macrophages (pMacs) were harvested via peritoneal lavage several days after i.p. injection of paraffin oil. The macrophages were stimulated with 50 μg/mL ox-LDL for 24 h after culture for 24–48 h.

### Immunoblotting Analysis

For immunoblotting, proteins were subjected to 10% SDS-PAGE separation and then transferred to PVDF membranes. The membranes were blocked with 5% non-fat milk in TBST for 1 h and incubated overnight at 4°C with primary antibodies. After incubation with secondary antibodies, relative expression was visualized using a ChemiDoc^TM^ Touch Imaging System (Bio-Rad).

### Real-Time PCR

RNA was extracted under RNase-free conditions using an E.Z.N.A. Total RNA Kit II (OMEGA Bio-tek) and reverse transcribed into cDNA with a PrimeScript^TM^ RT reagent kit (TaKaRa). Single-stranded cDNA was amplified via RT-PCR using TB Green (TaKaRa) on a QuantStudio 7 Flex Real-Time PCR System (Applied Biosystems). The quantified transcripts from the samples were normalized against *ACTB* gene expression.

### Oil Red O Staining Analysis

Cells were fixed with 4% paraformaldehyde (PFA) and then washed with PBS. After a rinse with isopropanol, the cells were stained with Oil Red O for 2 min and counterstained with hematoxylin. Cell morphology was observed using a microscope system (Olympus, Tokyo, Japan). The Oil Red O staining was quantified by measuring absorbance at 492 nm with a BIO-RAD Microplate reader (CA, United States) after extraction with isopropanol. For aorta staining, entire aortas were isolated and stained with Oil Red O for 1 h for en face analysis.

### Bodipy Staining

Cells were fixed in 4% PFA, permeabilized with 0.2% Triton X-100, and blocked with 10% BSA. The cells were then incubated with F4/80 antibody overnight at 4°C and with Alexa Fluor 555-conjugated secondary antibody (4417, Cell Signaling Technology) for 1 h at 37°C. Cell neutral lipids were stained with Bodipy, and cell nuclei were dyed with DAPI. Images were captured using an A1R-A1 confocal laser microscope system (Nikon, Tokyo, Japan).

### Intracellular Total Cholesterol and Cholesteryl Ester Measurement

Macrophage foam cells (1 × 10^6^) were washed with PBS and extracted with 200 μL of chloroform: isopropanol: NP-40 (7:11:0.1) in a microhomogenizer. After the cells were air dried and vacuumed, a cholesterol quantitation kit (MAK043, Sigma-Aldrich) was used to detect the total and free cholesterol; the concentration of cholesteryl ester = total cholesterol – free cholesterol. Protein content was detected using a BCA assay kit (Beyotime Biotechnology).

### Filipin Staining

A cholesterol cell-based detection assay kit (10009779, Cayman Chemical) was used for histochemical identification of cholesterol. Cells were incubated with Filipin III in the dark for 60 min and rapidly examined with a Cytation 5 Cell Imaging Multi-Mode Reader (BioTek) capable of excitation at 340–380 nm and measuring emission at 385–470 nm.

### Cholesterol Uptake Assay

DiI-labeled ox-LDL was used to trace cholesterol uptake. BMDMs were treated with DiI-ox-LDL (20 μg/mL) for 4 h at 37°C and washed several times. For fluorescence microscopy, cells were fixed with 4% PFA. Cell nuclei were dyed with DAPI and visualized using a confocal laser microscope system (Nikon, Tokyo, Japan). For flow cytometry, cells were detached from the plate and stained for the cell surface markers CD11b and F4/80 for 30 min. After several washes, the cells were analyzed with an LSR Fortessa flow cytometer (BD Biosciences, CA, United States), and the data were processed using FlowJo software.

### Cholesterol Efflux Assay

Cholesterol efflux was measured with a cholesterol efflux assay kit (ab196985, Abcam). BMDMs were incubated with Labeling Reagent + Equilibration Buffer mix containing ox-LDL (50 μg/mL) in the absence or presence of the LXR agonist GW3965 HCL (Selleck) at 2 μM as required. After overnight incubation, the cells were treated with either HDL (50 μg/mL) or ApoAI (10 μg/mL). Fluorescence intensity was quantified in the medium and in cells.

### RNA-Sequencing and Gene Expression Analysis

Total RNA was extracted from CD147^WT^ BMDMs and CD147^M–KO^ BMDMs using TRIzol (Invitrogen). Oligo(dT)-attached magnetic beads were used to purify the mRNA. Library construction and sequencing were performed by BGI (China). For data analysis, the sequencing data were filtered with SOAPnuke. Clean reads were aligned to the genome using HISAT2 (v2.0.4), and Bowtie2 (v2.2.5) was applied to map the clean reads to a coding gene set. Differential expression analysis was performed using DESeq2 (v1.4.5) with a *Q*-value ≤ 0.05. We performed Kyoto Encyclopedia of Genes and Genomes (KEGG) pathway analysis and Gene Set Enrichment Analysis (GSEA) with a Dr. Tom II system provided by BGI. For GSEA, the normalized enrichment score (NES) and false discovery rate (FDR) were used to quantify enrichment magnitude and statistical significance, respectively.

### Statistical Analysis

All experiments were performed independently at least three times. The results are presented as the mean ± SEM. The distribution of the data was checked using Shapiro-Wilk normality test. For parametric analysis, a two-tailed Student’s *t*-test was used for comparison between groups and one- or two-way ANOVA followed by a *post-hoc* Bonferroni’s test for multiple comparisons. For non-parametric analysis, a Mann-Whitney test was used to compare the values between the two groups and the Kruskal-Wallis test with *post-hoc* Dunn’s was used for multiple comparison data. Fisher’s exact test was used for contingency analysis. The degree of linearity was analyzed by Pearson’s correlation coefficient. All statistical analyses were performed with GraphPad Prism software (version 8) and SPSS software (version 25.0). *P*-values < 0.05 were considered to be statistically significant.

## Results

### CD147 Expression Is Increased in Mice and Human During Atherosclerosis

**FIGURE 1:**
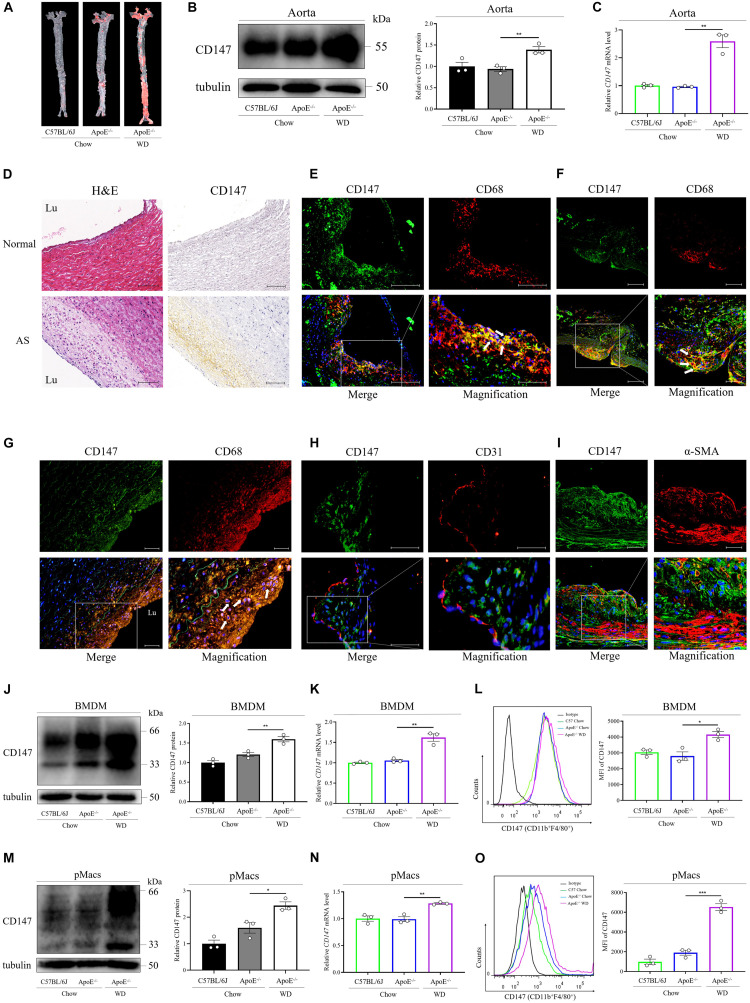
CD147 is upregulated in macrophage foam cells during atherosclerosis. **(A)** Atherosclerotic lesions in chow-fed C57BL/6J and *ApoE^–/–^* mice and WD-fed *ApoE^–/–^* mice were stained with Oil Red O. **(B)** CD147 expression in aortas of mice from each group was detected by western blotting. CD147 expression is presented relative to that of tubulin (right). **(C)** *CD147* mRNA levels were compared among the aortas of chow-fed C57BL/6J and *ApoE^–/–^* mice and WD-fed *ApoE^–/–^* mice. **(D)** Immunohistochemical staining for CD147 in healthy human arteries and atherosclerotic plaques after autopsy, also stained with H&E to show histological features. Scale bar, 100 μm. **(E–G)** Immunofluorescence staining for CD147 and CD68 in the aortic sinus **(E)** and aortic arches **(F)** of 16 w WD-fed *ApoE^–/–^* mice and in human atherosclerotic lesions **(G)**. (CD147, green; CD68, red; DAPI, blue; colocalization, yellow merge, see arrows). The scale bars in **(E)** are 100 μm and 50 μm, in **(F)** are 100 μm and 50 μm, in **G** is 50 μm. **(H,I)** Atherosclerotic plaques in WD-fed *ApoE^–/–^* mouse aortic sinus stained for CD147 and CD31 **(H)** or α-SMA **(I)**. The scale bars in **(H,I)** are 50 μm. **(J–O)** Western blotting **(J,M)**, RT-PCR **(K,N)**, or flow cytometry **(L,O)** of CD147 expression in CD11b^+^F4/80^+^ BMDMs **(J–L)** and pMacs **(M–O)** isolated from normal chow-fed C57BL/6J and *ApoE*^– /^*^–^* mice and WD-fed *ApoE*^–/^*^–^* mice. Right panel in **(J,M)**: CD147 expression is presented relative to that of tubulin. Right panel in **(L,O)**: quantification of the MFI of CD147 in each group. Data represent the mean ± SEM. of *n* = 3 biologically independent experiments. *P < 0.05, **P < 0.01, ***P < 0.001.

To determine the role of CD147 in atherosclerosis development, we first assessed its expression in the aorta of Western diet-fed *ApoE^–/–^* mice, a commonly used animal model of atherosclerosis. Compared with normal chow-fed C57BL/6J and *ApoE^–/–^* mice, the expression of CD147 protein and mRNA was more abundant in Western diet-fed *ApoE^–/–^* mice (Figures 1A–C and Supplementary Figure 1A). Importantly, examination of a human tissue microarray containing 21 human artery tissues and 8 atherosclerosis samples confirms a higher expression of CD147 in atherosclerosis (Figure 1D and Supplementary Figure 1B). These results suggest that increased CD147 expression is an important feature of mouse and human atherosclerotic lesions.

### CD147 Is Expressed on Macrophage Foam Cells in Atherosclerotic Plaques

To explore CD147 involvement in specific cell types during atherosclerosis, the atherosclerotic lesions of *ApoE^–/–^* mice that had been fed a Western diet for 16 weeks were examined via immunofluorescence staining. Double immunofluorescence staining revealed strong CD147 expression on macrophage foam cells (CD68-positive cells) in the aortic sinus (Figure 1E) and aortic arches (Figure 1F), where atherosclerotic plaques accumulate due to oscillatory shear stress. Moreover, colocalization of CD147 and CD68 was observed in human atherosclerotic plaques (Figure 1G). However, CD147 expression was not obviously present on the endothelial layer (CD31-positive cells) (Figure 1H) or vascular smooth muscle cells (α-SMA-positive cells) (Figure 1I), indicating that CD147 is specifically upregulated in atherosclerotic lesions that are rich in macrophages.

Furthermore, we isolated BMDMs (CD11b^+^F4/80^+^) from C57BL/6J and *ApoE^–/–^* mice that were fed either chow or a Western diet. As detected by western blotting (Figure 1J), RT-PCR (Figure 1K), and flow cytometry (Figure 1L), CD147 was expressed at a higher level on BMDMs from Western diet-fed *ApoE^–/–^* mice than on BMDMs from chow-fed mice. Similar results were shown in pMacs (CD11b^+^F4/80^+^) isolated from the above mice (Figures 1M–O). These data confirm increased CD147 expression on macrophages during atherosclerosis, which might be related to lipid accumulation and foam cell formation.

### Macrophage CD147 Is Upregulated by Ox-LDL

**FIGURE 2:**
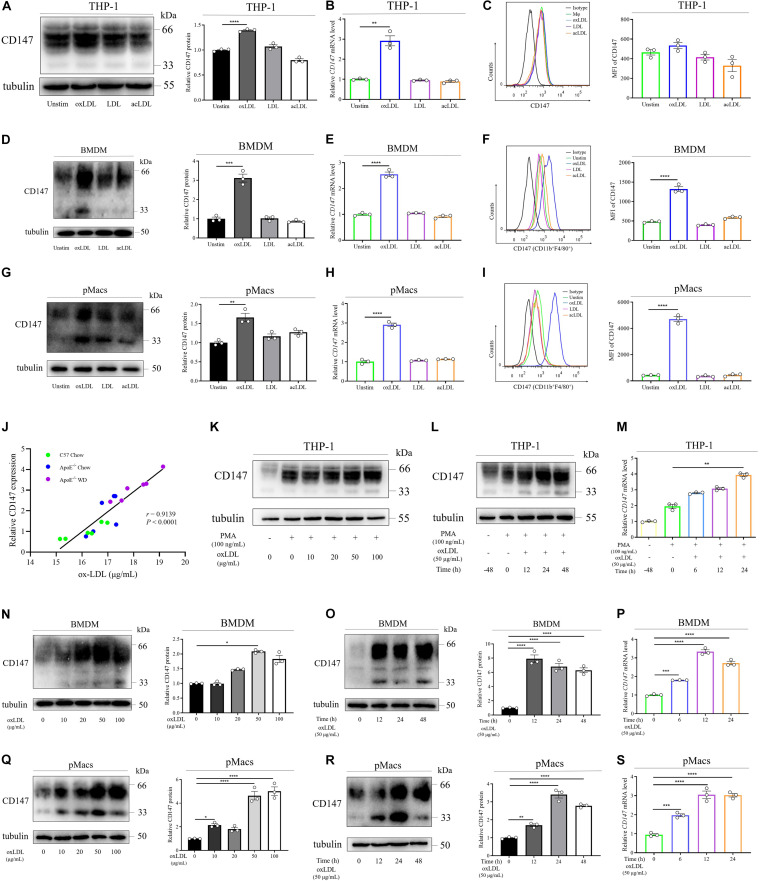
Macrophage CD147 is upregulated by ox-LDL. **(A–I)** CD147 expression was assessed by western blotting **(A,D,G)**, RT-PCR **(B,E,H)**, or flow cytometry **(C,F,I)** in THP-1-induced macrophages **(A–C)** and CD11b^+^F4/80^+^ BMDMs **(D–F)** and pMacs **(G–I)** incubated with ox-LDL, LDL, or ac-LDL (50 μg/mL) for 24 h. Right panel in **(A,D,G)**: CD147 expression is presented relative to that of tubulin (right). Right panel in **(C,F,I)**: quantification of the MFI of CD147 in each group. **(J)** The relative expression of CD147 by IHC was positively correlated with the serum level of ox-LDL (*r* = 0.9139, *P* < 0.0001). **(K–S)** Western blot analysis of CD147 protein levels in THP-1-induced macrophages **(K)**, BMDMs **(N)**, and pMacs **(Q)** treated with different doses of ox-LDL for 24 h. RT-PCR analysis of *CD147* mRNA levels in THP-1-induced macrophages **(L)**, BMDMs **(O)**, and pMacs **(R)** exposed to 50 μg/mL ox-LDL for the indicated time. Flow cytometry in THP-1-induced macrophages **(M)**, BMDMs **(P)**, and pMacs **(S)** after treatment of 50 μg/mL ox-LDL for the indicated time. Data represent the mean ± SEM. of *n* = 3 biologically independent experiments. *P < 0.05, **P < 0.01, ***P < 0.001, ****P < 0.0001.

To further evaluate the effects of atherosclerotic stimuli on CD147 expression in macrophages, we used various lipoproteins, including ox-LDL, LDL, and ac-LDL, to treat human THP-1-induced macrophages (Figures 2A–C), primary BMDMs (Figures 2D–F), and pMacs (Figures 2G–I) from C57BL/6J mice. Western blotting (Figures 2A,D,G), RT-PCR (Figures 2B,E,H), and flow cytometric (Figures 2C,F,I) analyses showed that CD147 expression was significantly upregulated only by ox-LDL and not by native LDL or ac-LDL. Furthermore, high CD147 expression was highly related to high level of ox-LDL in serum of mice, as verified by Pearson’s correlation test (Figure 2J and Supplementary Figure 1C). These findings indicate that CD147 expression on macrophage foam cells could be regulated by ox-LDL.

As macrophage foam cell formation is a central step in atherosclerosis development, we next validated the involvement of CD147 in foam cell formation. We found a dose-dependent and time-dependent promotion of the CD147 protein and mRNA levels during the process by which human THP-1 monocytes differentiate into macrophages under PMA stimulation and further transform into foam cells by ox-LDL treatment (Figures 2K–M). Consistently, in mouse primary BMDMs (Figures 2N–P), and pMacs (Figures 2Q–S), both protein and mRNA levels of CD147 were remarkably upregulated in a dose-dependent and time-dependent manner when incubated with ox-LDL. Collectively, these results provide solid evidence that macrophage CD147 is not only enhanced in atherosclerotic plaques *in vivo*, but it is also upregulated by ox-LDL in foam cell formation *in vitro*, indicating that CD147 might play a role in atherosclerosis development.

### CD147 Upregulation by Ox-LDL Is Mediated by PI3K/Akt/mTOR Signaling

**FIGURE 3:**
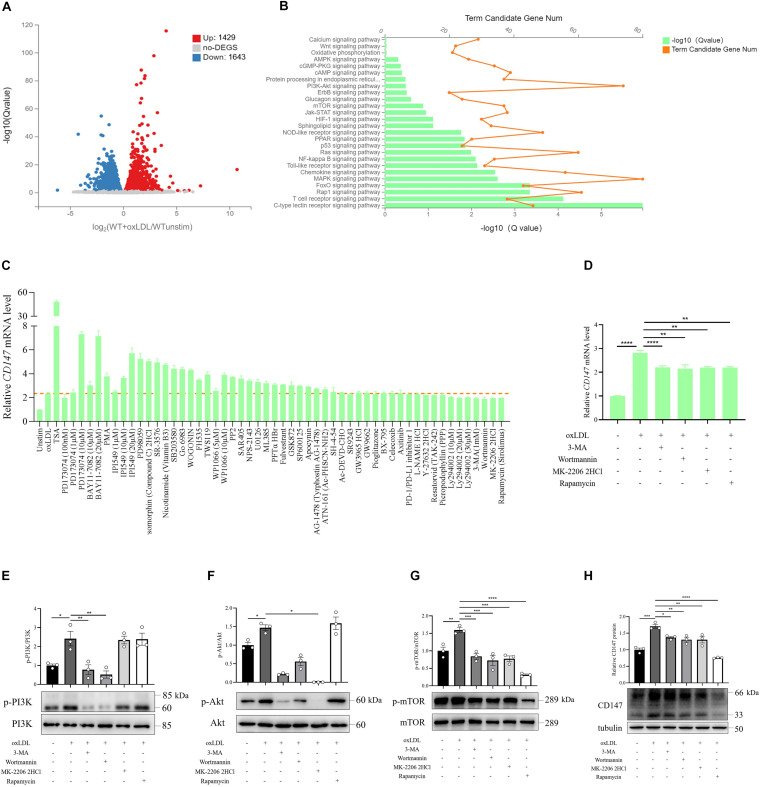
Compound screening identifies PI3K/Akt/mTOR signaling as a regulator of CD147 expression induced by ox-LDL in macrophages. **(A)** WT BMDMs treated with or without ox-LDL (50 μg/mL) for 24 h were analyzed by RNA sequencing. Identification of DEGs is illustrated in a volcano plot. **(B)** KEGG pathway enrichment histogram showing genes involved in foam cell formation based on the upregulated DEGs. **(C)** Overview of a large compound screening by RT-PCR showing *CD147* mRNA levels in BMDMs incubated for 1 h with one of 46 potential compounds targeting 26 candidate signaling pathways and then treated with ox-LDL (50 μg/mL) for 24 h. **(D)** RT-PCR analysis of CD147 mRNA levels in BMDMs treated with ox-LDL (50 μg/mL) for 24 h in the presence or absence of 3-MA (1 mM), wortmannin (100 nM), MK-2206 2HCl (1 μM), or Rapamycin (100 nM). **(E–H)** Western blot analysis of phosphorylated (p-) and total PI3K, Akt, and mTOR and CD147 in BMDMs exposed to 50 μg/mL ox-LDL for 24 h in the presence or absence of 3-MA (1 mM), wortmannin (100 nM), MK-2206 2HCl (1 μM), or Rapamycin (100 nM). Data represent the mean ± SEM. of n ≥ 3 biologically independent experiments. *P < 0.05, **P < 0.01, ***P < 0.001, ****P < 0.0001.

To investigate the mechanism of CD147 upregulation on macrophage foam cells, we performed RNA-seq in unstimulated and ox-LDL-treated WT BMDMs. In total, 3,072 differentially expressed genes (DEGs) were identified, including 1,429 upregulated and 1,643 downregulated genes in ox-LDL-treated BMDMs (Figure 3A). We performed KEGG pathway enrichment analysis for all the DEGs and selected the signaling pathways related to foam cell formation and atherosclerosis for further analysis (Figure 3B). According to Figure 3B, forty-six potential compounds targeting 26 candidate signaling pathways were customized to screen the signaling implicated in CD147 upregulation on macrophage foam cells. High-throughput compound screening showed that the upregulation of *CD147* mRNA in ox-LDL-treated BMDMs was inhibited by 3-methyladenine (3-MA), wortmannin, and Ly294002 (PI3K inhibitors); MK-2206 2HCl (an Akt inhibitor); and Rapamycin (an mTOR inhibitor) (Figures 3C,D), strongly indicating that the PI3K/Akt/mTOR pathway may contribute to CD147 regulation. However, NF-κB inhibitors, such as BAY 11-7082 and pyrrolidine dithiocarbamate (PDTC), did not effectively block CD147 upregulation in foam cells in a dose gradient experiment, even promoting a striking upregulation of CD147, which followed the same trend as the NF-κB inducer PMA (Figure 3C). Moreover, CD147 protein level was not decreased when NF-κB inhibitor was treated in ox-LDL-induced macrophage foam cells isolated from both BMDM and pMacs (Supplementary Figure 1D). Eventually, four inhibitors targeting PI3K/Akt/mTOR signaling were chosen for additional evaluation. The phosphorylation of PI3K, Akt, and mTOR was increased in ox-LDL-treated BMDMs (Figures 3E–G). Notably, Rapamycin was shown to be the most potent inhibitor, significantly suppressing mTOR signaling and inducing the greatest reduction in ox-LDL-induced CD147 protein upregulation compared to PI3K inhibitors and the Akt inhibitor (Figures 3G,H). These data suggest that ox-LDL stimulates macrophage foam cells, resulting in upregulation of CD147, and this process is mediated by PI3K/Akt/mTOR signaling, especially mTOR, but not by NF-κB.

### Macrophage-Specific *CD147* Knockout Inhibits Foam Cell Formation

**FIGURE 4:**
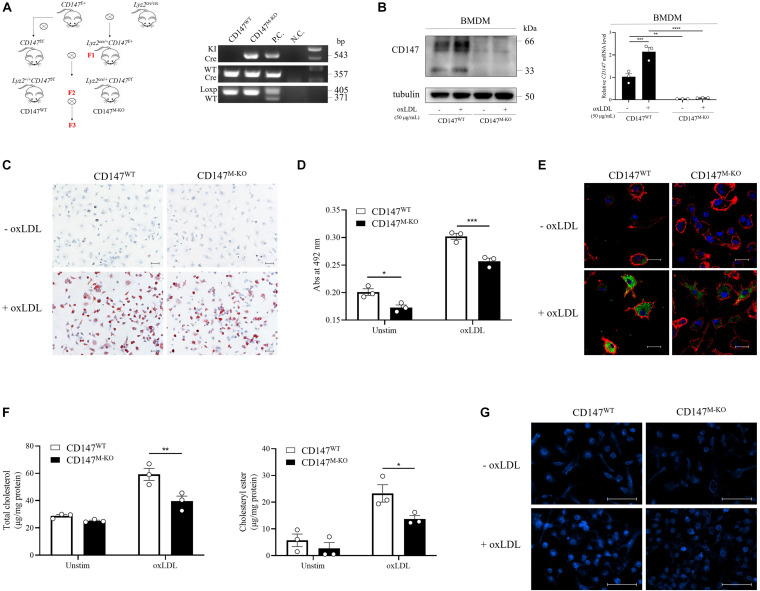
Macrophage-specific *CD147* knockout inhibits foam cell formation. **(A)** Generation of macrophage-specific *CD147* knockout mice (*Lyz2*^cre/+^*CD147*^f/f^, namely, CD147^M–KO^ mice) is illustrated by the mating scheme. The control WT littermates were *Lyz2*^+/+^*CD147*^f/f^, namely, CD147^WT^ mice. PCR analysis of genomic DNA showed the genotyping. The fragments from top to bottom are the KI *Cre* gene, WT *Cre* gene, and floxed and WT *CD147* gene. Genomic DNA from *Lyz2*^cre/+^ mice was used as the P.C. for *Cre* analysis. Genomic DNA from *CD147*^f/+^ mice was used as the P.C. for *CD147* analysis. H_2_O was used as the N.C. **(B)** Characterization of BMDMs isolated from CD147^WT^ or CD147^M–KO^ mice via western blotting and RT-PCR. **(C)** Representative images of Oil Red O staining of CD147^WT^ or CD147^M–KO^ BMDMs that were incubated with or without ox-LDL (50 μg/mL) for 24 h. The scale bar is 50 μm. **(D)** For quantification, Oil Red O absorbance was measured at 492 nm after extraction with isopropanol. **(E)** BMDMs from CD147^WT^ or CD147^M–KO^ mice stimulated with or without ox-LDL (50 μg/mL) for 24 h were stained with Bodipy (lipids, green), F4/80 (macrophages, red), and DAPI (nuclei, blue) and examined via confocal microscopy. The scale bar is 20 μm. **(F)** The total cholesterol and cholesteryl ester contents were determined with a coupled enzyme assay. **(G)** Representative images of Filipin staining of BMDMs from CD147^WT^ or CD147^M–KO^ mice treated with or without ox-LDL (50 μg/mL) for 24 h. The scale bar is 100 μm. Data represent the mean ± SEM. of *n* = 3 biologically independent experiments. *P < 0.05, **P < 0.01, ***P < 0.001, ****P < 0.0001.

To identify the role that macrophage CD147 plays during atherosclerosis development, we probed whether CD147 participates in foam cell formation. First, we generated macrophage-specific *CD147* knockout mice (*Lyz2*^cre/+^*CD147*^f/f^) using the Cre/Loxp system (Figure 4A). Western blotting and RT-PCR analyses confirmed the characterization of BMDMs isolated from *Lyz2*^+/+^*CD147*^f/f^ (CD147^WT^) and *Lyz2*^cre/+^*CD147*^f/f^ (CD147^M–KO^) mice (Figure 4B). CD147^WT^ and CD147^M–KO^ BMDMs were incubated with ox-LDL to induce foam cell formation. Oil Red O staining revealed that lipid accumulation was alleviated in CD147^M–KO^ macrophage foam cells compared with CD147^WT^ macrophage foam cells (Figure 4C). Subsequently, we extracted intracellular Oil Red O with isopropanol and determined the Oil Red O centration by measuring its absorbance at 492 nm (Figure 4D). In accordance with these results, Bodipy and F4/80 staining also showed a reduction in lipid deposition in CD147^M–KO^ macrophage foam cells (Figure 4E). Moreover, an intracellular cholesterol assay showed that the total cholesterol and cholesteryl ester contents were decreased in CD147^M–KO^ macrophage foam cells (Figure 4F). We also performed Filipin staining in ox-LDL-treated macrophages for histochemical identification. As shown in Figure 4G, under unstimulated conditions, the vast majority of total cellular cholesterol was present at the plasma membrane. After ox-LDL treatment, cholesterol droplets principally accumulated in intracellular sites. CD147^M–KO^ macrophages showed a remarkable reduction in Filipin staining in response to ox-LDL compared with their counterparts from CD147^WT^ mice. These findings demonstrate that macrophage-specific *CD147* knockout markedly reduces the capacity of macrophages to form foam cells.

### Macrophage-Specific *CD147* Knockin Promotes Foam Cell Formation

**FIGURE 5:**
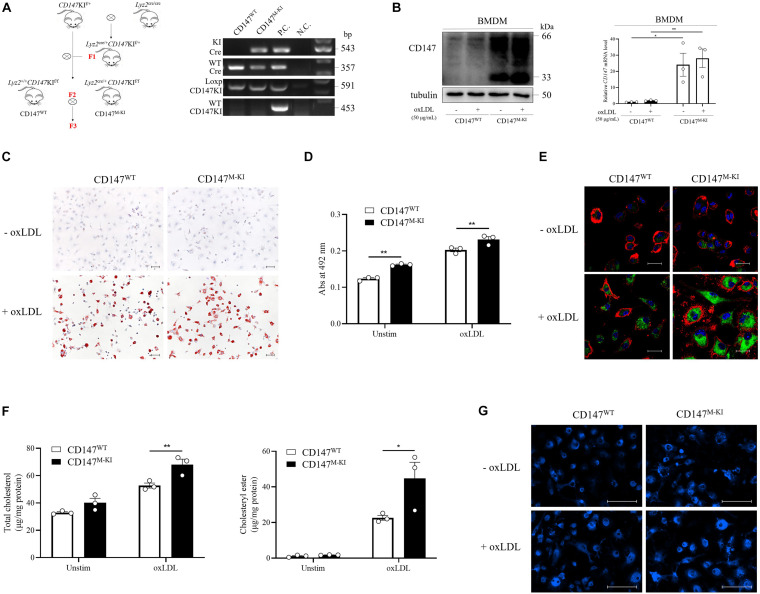
Macrophage-specific *CD147* knockin promotes foam cell formation. **(A)** Generation of macrophage-specific *CD147* knockin mice (*Lyz2*^cre/+^*CD147*KI^f/f^, namely, CD147^M–KI^ mice) is illustrated by the mating scheme. The control WT littermates were *Lyz2*^+/+^*CD147*KI^f/f^, namely, CD147^WT^ mice. PCR analysis of genomic DNA showed the genotyping. The fragments from top to bottom are the KI *Cre* gene, WT *Cre* gene, floxed *CD147*KI gene and WT *CD147*KI gene. Genomic DNA from *Lyz2*^cre/+^ mice was used as the P.C. for *Cre* analysis. Genomic DNA from *CD147*KI^f/+^ mice were used as the P.C. for *CD147* analysis. H_2_O was used as the N.C. **(B)** Characterization via western blotting and RT-PCR of BMDMs with or without ox-LDL treatment isolated from CD147^WT^ or CD147^M–KI^ mice. **(C)** Representative images of Oil Red O staining of CD147^WT^ or CD147^M–KI^ BMDMs that were incubated with or without ox-LDL (50 μg/mL) for 24 h. The scale bar is 50 μm. **(D)** For quantification, Oil Red O absorbance was measured at 492 nm after extraction with isopropanol. **(E)** BMDMs from CD147^WT^ or CD147^M–KI^ mice stimulated with or without ox-LDL (50 μg/mL) for 24 h were stained with Bodipy (lipids, green), F4/80 (macrophages, red), and DAPI (nuclei, blue) and examined via confocal microscopy. The scale bar is 20 μm. **(F)** The total cholesterol and cholesteryl ester contents were determined with a coupled enzyme assay. **(G)** Representative images of Filipin staining of BMDMs from CD147^WT^ or CD147^M–KI^ mice treated with or without ox-LDL (50 μg/mL) for 24 h. The scale bar is 100 μm. Data represent the mean ± SEM. of *n* = 3 biologically independent experiments. *P < 0.05, **P < 0.01.

To confirm the effect of CD147 on promotion of foam cell formation, we further generated macrophage-specific CD147 knockin (*Lyz2*^cre/+^*CD147*KI^f/f^) using the Cre/Loxp (Figure 5A). The levels of CD147 protein and mRNA confirmed the mouse construction (Figure 5B). BMDMs isolated from *Lyz2*^+/+^*CD147*KI^f/f^ (CD147^WT^) and *Lyz2*^cre/+^*CD147*KI^f/f^ (CD147^M–KI^) mice were incubated with ox-LDL. In line with expectations, macrophage-restricted CD147 overexpression led to a remarkable increase in foam cell formation, as demonstrated by Oil Red O staining (Figure 5C) and its intracellular concentration (Figure 5D) in CD147^M–KI^ macrophage foam cells. This was further verified by the dramatic elevation in CD147^M–KI^ macrophage foam cells double positive for Bodipy and F4/80 (Figure 5E). Consistently, the total cholesterol and cholesteryl ester levels were highly improved in the course of macrophage-restricted CD147 overexpression (Figure 5F). This phenomenon was confirmed by Filipin staining, which revealed augmented cholesterol droplets inside the CD147^M–KI^ macrophage foam cells (Figure 5G). Taken together, these data strongly indicate that CD147 powerfully facilitates foam cell formation and that inhibition of CD147 exerts a protective effect against this process.

### Macrophage CD147 Accelerates Ox-LDL Uptake but Does Not Affect Cholesterol Efflux

**FIGURE 6:**
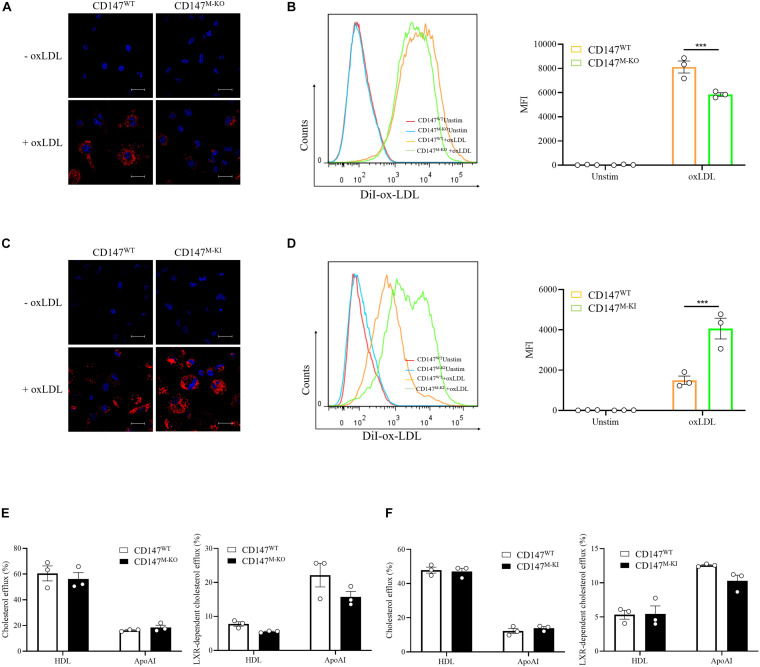
Macrophage CD147 regulates foam cell formation by mediating ox-LDL uptake. **(A,B)** BMDMs from CD147^WT^ or CD147^M–KO^ mice were examined using either confocal microscopy **(A)** or flow cytometry **(B)** after treatment with DiI-oxLDL (20 μg/mL) for 4 h. The scale bar is 20 μm. Right panel: Quantification of MFI of DiI-oxLDL in CD11b^+^F4/80^+^ BMDMs. **(C,D)** BMDMs from CD147^WT^ or CD147^M–KI^ mice were examined using either confocal microscopy **(C)** or flow cytometry **(D)** after treatment with DiI-oxLDL (20 μg/mL) for 4 h. The scale bar is 20 μm. Right panel: Quantification of MFI of DiI-oxLDL in CD11b^+^F4/80^+^ BMDMs. **(E,F)** A cholesterol efflux assay kit was used to detect LXR-independent and -dependent cholesterol efflux to HDL or ApoAI in BMDMs isolated from CD147^M–KO^ **(E)** or CD147^M–KI^ **(F)** mice and their CD147^WT^ littermates. Data represent the mean ± SEM. of *n* = 3 biologically independent experiments. ***P < 0.001.

Foam cell formation is thought to be regulated by the balance between uptake of modified lipoproteins and cholesterol efflux (Li and Glass, 2002). To determine how macrophage CD147 affects foam cell formation, we first used DiI-labeled ox-LDL to trace ox-LDL uptake. As detected by immunofluorescence (Figure 6A) and flow cytometry (Figure 6B), ox-LDL uptake by BMDMs isolated from CD147^M–KO^ mice was significantly mitigated compared with their counterparts from CD147^WT^ mice. The role of CD147 in ox-LDL uptake was further substantiated in CD147^M–KI^ macrophages, as indicated by the uncontrolled increase in fluorescence intensity observed in immunofluorescence experiments (Figure 6C) and the approximately threefold promotion of mean fluorescence intensity in flow cytometric analysis (Figure 6D). Collectively, attenuated intracellular ox-LDL uptake could account for suppressed foam cell formation in CD147^M–KO^ macrophages, whereas uptake is increased in CD147^M–KI^ macrophages.

Next, we used fluorescence-labeled cholesterol to examine cholesterol efflux. However, there was no obvious difference in the LXR agonist GW3965 HCl-independent or -dependent cholesterol efflux to lipid-poor HDL or ApoAI between CD147^WT^ and CD147^M–KO^ BMDMs (Figure 6E) or between CD147^WT^ and CD147^M–KI^ BMDMs (Figure 6F). Thus, macrophage CD147 facilitates foam cell formation through increased ox-LDL uptake.

### CD147 May Promote Macrophage Cholesterol Uptake through Induction of CD36

**FIGURE 7:**
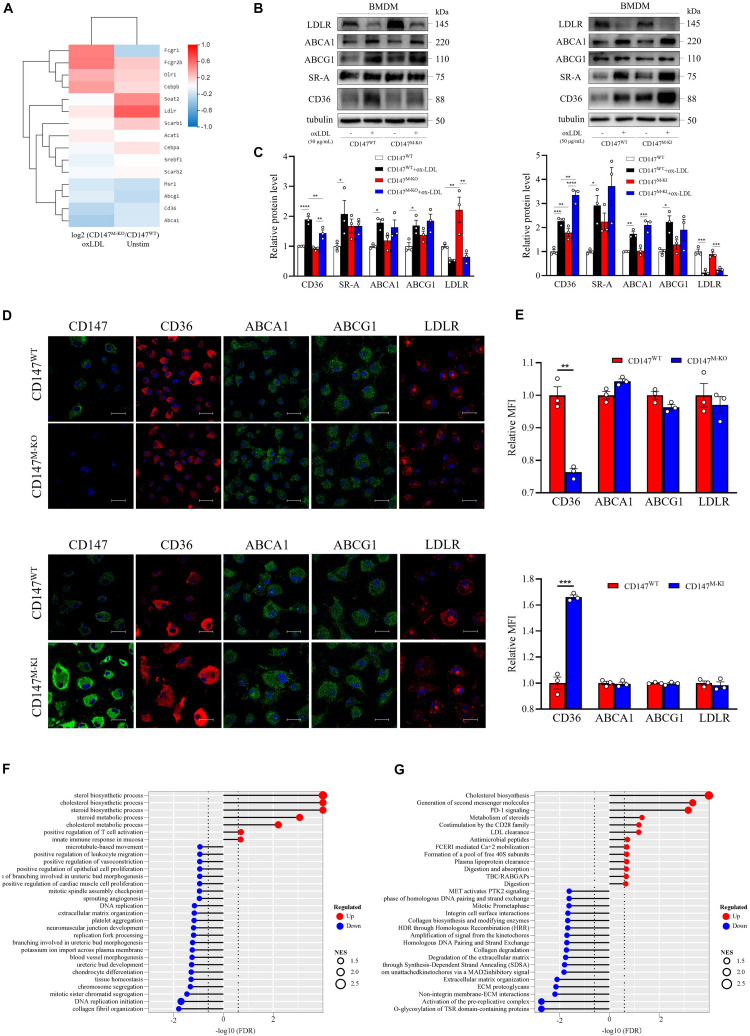
Macrophage-specific *CD147* deficiency diminishes CD36 expression and may exert other protective effects in atherosclerosis. **(A)** BMDMs from CD147^WT^ and CD147^M–KO^ treated with or without ox-LDL (50 μg/mL) for 24 h were analyzed by RNA sequencing. The selected genes involved in foam cell formation were graphed in a heatmap. **(B)** Western blot analysis of LDLR, ABCA1, ABCG1, SR-A, and CD36 in BMDMs isolated from CD147^M–KO^ and CD147^M–KI^ mice and their respective CD147^WT^ littermates incubated with or without ox-LDL (50 μg/mL) for 24 h. **(C)** Quantification of protein levels of molecules in **(B)** relative to tubulin. **(D)** Immunofluorescence staining of CD147, CD36, ABCA1, ABCG1, and LDLR in BMDMs isolated from CD147^M–KO^ and CD147^M–KI^ mice and their respective CD147^WT^ littermates treated with ox-LDL (50 μg/mL) for 24 h. Scale bar, 20 μm. **(E)** Quantification of the MFI of **(D)** by means of the Image-Pro Plus 6.0 software. **(F,G)** GESA GO enrichment analysis for biological process **(F)** and GESA reactome enrichment analysis **(G)** are shown as lollipop charts. Statistical significance in gene sets was defined as an FDR < 0.25; NES represents enrichment magnitude and is shown as the size of the point. The −log_10_ (FDR) of the enrichment is shown on the *x*-axis; the enriched gene set is shown on the *y*-axis; and the color of points represent the fraction of gene sets that are significantly upregulated or downregulated. Data represent the mean ± SEM. of *n* = 3 biologically independent experiments. *P < 0.05, **P < 0.01, ***P < 0.001, ****P < 0.0001.

To determine the mechanism involved in enhanced cholesterol uptake by macrophage CD147, we collected unstimulated or ox-LDL-treated BMDMs from CD147^WT^ and CD147^M–KO^ mice and performed RNA-seq. We selected a variety of genes involved in foam cell formation. and the results are illustrated in a heatmap (Figure 7A). The expression of the major scavenger receptor *CD36* was downregulated by macrophage-specific *CD147* knockout in both unstimulated and ox-LDL-treated macrophages. Importantly, the CD36 protein level was decreased in unstimulated CD147^M–KO^ macrophages, and the decrease was more pronounced in ox-LDL-treated macrophage foam cells isolated from CD147^M–KO^ mice compared with those from CD147^WT^ littermates. The opposite effect was observed in BMDMs isolated from CD147^M–KI^ mice (Figures 7B–E). Noticeably, LDLR mRNA and protein expression was remarkably promoted in untreated CD147^M–KO^ macrophages. However, the expression levels of the other scavenger receptor SR-A required for cholesterol uptake; ATP-binding cassette (ABC) transporters for cholesterol efflux, including ABCA1 and ABCG1; and enzymes responsible for cholesterol esterification were comparable after deletion or overexpression of CD147 (Figures 7B–E). These results suggest that the alteration in ox-LDL uptake in macrophages induced by targeting of CD147 is possibly due to regulation of the scavenger receptor CD36.

### Macrophage *CD147* Deficiency May Protect Against Atherosclerosis in Versatile Aspects

Further GSEA Gene Ontology (GO) analysis confirmed the role of CD147 in cholesterol uptake, where lipid metabolism, including the cholesterol biosynthetic process and cholesterol metabolic process, was found to be enriched in the CD147^M–KO^ macrophages (Figure 7F). Similar results were obtained with GSEA reactome enrichment analysis, where cholesterol biosynthesis was ranked highest (Figure 7G). Moreover, LDL clearance and plasma lipoprotein clearance were upregulated in CD147^M–KO^ macrophages, which might block foam cell formation at its source. Furthermore, related genes involved in these two processes were selected and delineated with heatmaps (Supplementary Figure 1E). Additionally, GSEA GO process enrichment analysis showed that platelet aggregation was downregulated, and the reactome revealed that collagen degradation was decreased when macrophage *CD147* was deficient. The heatmaps are presented in Supplementary Figure 1E. All these results suggest that ablation of macrophage CD147 may exert various atheroprotective effects.

## Discussion

Increasing studies suggest that atherosclerosis is a chronic inflammatory disease of large and medium arteries, with characteristic foam cell formation due to cholesterol accumulation (Chen et al., 2020; Ma et al., 2020). Therefore, inhibiting foam cell formation is a fundamental step in slowing the progression of atherosclerotic plaques. Despite decades of research, the underlying mechanisms of foam cell formation regulation still must be fully elucidated. Here, we first report that CD147 is involved in the pathogenesis of atherosclerosis by stimulating foam cell formation, as supported by several lines of evidence: (1) CD147 expression is increased in mouse and human atherosclerotic plaques; (2) CD147 is primarily expressed on macrophage foam cells in atherosclerotic lesions but not on endothelial cells or vascular smooth muscle cells; (3) CD147 in human THP-1-induced macrophages and mouse primary BMDMs and pMacs can be upregulated by ox-LDL, but not by other LDLs, such as native LDL or ac-LDL; (4) CD147 upregulation driven by ox-LDL is mediated by PI3K/Akt/mTOR signaling, especially mTOR; (5) macrophage-specific *CD147* knockout inhibits foam cell formation, whereas macrophage-specific *CD147* knockin promotes foam cell formation; (6) deletion or overexpression of *CD147* in macrophages ameliorates or accelerates ox-LDL uptake, respectively, but does not affect cholesterol efflux; (7) CD147 promotes cholesterol uptake possibly through the induction of the scavenger receptor CD36; and (8) Macrophage CD147 may participate in various processes to protect against atherosclerosis, including LDL clearance, plasma lipoprotein clearance, platelet aggregation, and collagen degradation.

*ApoE*^–/^*^–^* is an excellent mouse model of hyperlipidemia and atherosclerosis. It is well accepted that the serum levels of total cholesterol and LDL-C are increased in chow diet-fed *ApoE*^–/^*^–^* mice, which are more remarkably promoted in Western diet-fed *ApoE*^–/^*^–^* mice (Piedrahita et al., 1992; Moghadasian et al., 2001). Our serum lipid profile data reach the same conclusion. Notably, in our study, CD147 mRNA and protein expression is not elevated in aorta of chow diet-fed *ApoE*^–/^*^–^* mice as compared to C57BL/6J mice. The possible reason accounting for this seemingly surprising phenomenon is that 16 w chow diet is not enough for CD147 expression elevation, indicating that the CD147 increase is a later-stage reaction occurred in medium and advanced atherosclerosis, but not in hyperlipidemia or early atherosclerosis. In addition, as the western blot statistics shown in Figures 1J,M, CD147 protein levels tend to increase in primary BMDMs and pMacs from chow diet-fed *ApoE*^–/–^ mice compared to those from C57BL/6J mice. Although oxLDL accumulates in the intima of aorta of *ApoE*^–/–^ mice with chow diet, CD147 expression in aorta of those mice does not show an increase presumably due to its complicated components, suggesting the specific expression of CD147 in macrophages.

The evidence that whether CD147 is expressed in arterial smooth muscle cells and endothelial cells of atherosclerotic lesions remains controversial. Major et al. (2002) have demonstrated that CD147 is localized to lesion areas of the human atheromas, where the areas of strong CD147 protein expression coincide with macrophage areas and not to smooth muscle cell areas. In contrast, Ramirez-Carracedo and colleagues (Ramirez-Carracedo et al., 2018) have reported CD147 expression in foam cells, smooth muscle cells, and endothelial cells of vascularized plaques. However, CD147 expression in both studies is only determined by immunohistochemistry, which is lack of specific markers for indicating cell types. In our study, double immunofluorescence staining is examined via confocal microscopy to explore CD147 involvement in specific cell types in atherosclerotic lesions. Our results suggest that CD147 is specifically upregulated in atherosclerotic lesions that are rich in macrophages, whereas it is not obviously present on the endothelial layer (CD31-positive cells) or vascular SMCs (α-SMA-positive cells).

We have found that there is a huge difference existed in the basal level and fold of changes of CD147 in BMDMs and THP-1 cells upon ox-LDL treatment. Primary BMDMs are isolated from bone marrow cells in mouse femurs and tibias, which differentiate into macrophages after 7 day culture under M-CSF induction, subsequently forming foam cells when incubated with ox-LDL (Choi et al., 2015; Fan et al., 2019). Our data show that the basal level of CD147 in BMDMs is relative low and boosted in foam cells (Figure 2N). However, the human monocyte THP-1 cell line is derived from patient with acute monocytic leukemia and can differentiate into macrophages upon PMA stimulation and form foam cells when treated with ox-LDL (Liang et al., 2019). It is in monocytes that CD147 has a relative low basal level and in macrophages that CD147 shows a remarkable increase. However, CD147 level is slightly increased in the forming process from macrophages to foam cells (Figure 2K). On the other hand, there is an intrinsic difference between primary cells and cell lines. Therefore, we illustrate that CD147 level is increased during foam cell formation using both mouse primary BMDMs and pMacs and human THP-1 cell line.

It has been recognized that NF-κB plays an important role in foam cell formation and CD147 regulation. NF-κB can be activated by ox-LDL in macrophages in a CD36-dependent manner (Janabi et al., 2000). Bay 11-7082, an inhibitor of NF-κB activation, is shown to block macrophage ox-LDL uptake (Etzion et al., 2009). Accumulating studies show that CD147 is implicated in NF-κB signaling through promotion of NF-κB activation, which participates in diverse processes, including apoptosis and inflammation (Schmidt et al., 2008; Zhai et al., 2016). Therefore, we assume that NF-κB could regulate CD147 expression in ox-LDL-induced macrophage foam cells. However, BAY 11-7082 does not effectively block CD147 upregulation induced by ox-LDL (50 mg/mL) in foam cells in a dose gradient experiment, even promoting a striking upregulation of CD147, which follows the same trend as the NF-κB activator PMA (Figure 3C). Another NF-κB inhibitor, PDTC, results in foam cell death at concentrations of 50 μM, 10 μM, and even 1 μM. To investigate the mechanism of CD147 upregulation on macrophage foam cells, we perform RNA-seq and high-throughput compound screening according to KEGG enrichment analysis. Remarkably, the upregulation of CD147 mRNA and protein in ox-LDL-treated BMDMs is inhibited by 3-MA and wortmannin (PI3K inhibitors), MK-2206 2HCl (an Akt inhibitor), and Rapamycin (an mTOR inhibitor) (Figures 3C–H), strongly indicating that the PI3K/Akt/mTOR pathway contributes to CD147 regulation. Rapamycin is shown to be the most potent inhibitor of mTOR signaling, leading to the greatest reduction in ox-LDL-induced CD147 protein upregulation compared to PI3K inhibitors and the Akt inhibitor (Figure 3G). These data suggest that ox-LDL stimulation of macrophage foam cells leads to upregulation of CD147, which is mediated by PI3K/Akt/mTOR signaling, especially mTOR, but not NF-κB. However, how does PI3K/Akt/mTOR signaling regulate CD147 expression and function in foam cell formation still needs further investigation. Remarkably, the involvement of CD147 and transcription factors has been reported by our previous work, providing future research directions. The transcription factor Slug is the CD147 upstream mediator in epithelial-mesenchymal transition during hepatocellular carcinoma progression, the signaling cascade of which is TGF-β/PI3K/Akt/GSK3β/Snail/Slug/CD147 (Wu et al., 2011). In addition, dual-luciferase reporter assay and chromatin immunoprecipitation results have shown that CD147 can be transcriptionally regulated by Smad4 in liver fibrosis (Li H.Y. et al., 2015). Furthermore, transcription factor Sp1, a member of the zinc-finger Sp family of proteins including the Kruppel-like factor family, regulates CD147 expression in human lung cancer (Kong et al., 2010).

CD147 induces elevated intracellular accumulation of cholesteryl ester and foam cell formation, as demonstrated by macrophage-specific *CD147* knockout and macrophage-restricted *CD147*-overexpressing mice. Foam cell formation is thought to be regulated by the balance between uptake of modified lipoproteins and cholesterol efflux. CD36 is accepted as an important orchestrator in promotion of cholesterol uptake. Ox-LDL interaction with CD36 triggers both pro-atherogenic and pro-inflammatory responses (Han et al., 2004; Park, 2014). Our study proposes a novel mechanism underlying hyperlipidemia-induced formation of macrophage foam cells. We first demonstrate that the role of macrophage CD147 in atherosclerosis might involve promotion of ox-LDL-induced CD36-dependent cholesterol uptake and foam cell formation. These data add CD147 to the CD36 axis in the obscure network that regulates atherosclerosis development. However, the detailed mechanisms by which CD147 participates in ox-LDL-induced CD36 upregulation need to be further investigated. As a pro-inflammatory molecule, whether CD147 also plays a role in pro-inflammatory responses triggered by ox-LDL and CD36 is still unclear.

Apart from intracellular cholesterol metabolism, CD147 may be a critical target for protecting against atherosclerosis in other aspects. LDL-C is regarded as the most atherogenic form of cholesterol. Statins, the cornerstone for primary and secondary prevention of atherothrombosis, are prescribed with the aim of lowering LDL-C (Weitz and Fazio, 2019). Our data show that LDL clearance and plasma lipoprotein clearance were upregulated in CD147^M–KO^ macrophages, suggesting that CD147 may be a promising therapeutic target in atherothrombosis. Retention of foam cells in the intimal layer of arteries results in the formation of fatty streaks, which evolve into atherosclerotic plaques consisting of a lipid-rich necrotic core and a fibrous cap. Degradation of the extracellular matrix by MMPs contributes to thinning of the fibrous cap, which makes the plaque susceptible to rupture (Weitz and Fazio, 2019). Our GSEA reactome enrichment analysis shows that macrophage-specific *CD147* knockout contributes to decreased collagen degradation. CD147, known as an extracellular MMP inducer, can induce MMP production. Inhibition of MMPs increases collagen expression, promoting a fibrotic response, and subsequent stabilization of existing plaques. Atherosclerosis is usually silent until atherosclerotic plaque disruption triggers platelet aggregation and coagulation activation, culminating in the formation of platelet-rich thrombi that obstruct blood flow; this condition is known as atherothrombosis and eventually leads to cardiovascular events (Chan and Weitz, 2019). Our GSEA GO process enrichment analysis shows that platelet aggregation is suppressed under macrophage-specific *CD147* deficiency. In fact, CD147 has been reported as a novel receptor on platelets (Schmidt et al., 2008). In addition, CD147-mediated PI3K/Akt signaling activated by extracellular cyclophilin A can contribute to increased adhesion and thrombus formation (Seizer et al., 2015). Therefore, abolishing CD147 may have a protective effect, slowing atherothrombosis and avoiding cardiovascular events. Overall, macrophage-specific CD147 deletion may protect against atherosclerosis in versatile aspects, including lowering LDL-C, improving plaque stability, and preventing atherothrombosis.

In summary, we report for the first time that CD147 plays a pro-atherogenic role in facilitating ox-LDL uptake and foam cell formation. Our findings provide a foundation for use of CD147 as a therapeutic target for atherosclerosis.

## Data Availability Statement

Our RNA-seq original sequence data have been submitted to the database of the NCBI Sequence Read Archive (http://trace.ncbi.nlm.nih.gov/traces/sra) under the accession number: PRJNA665796.

## Ethics Statement

The animal study was reviewed and approved by the Animal Care and Use Committee of the Fourth Military Medical University.

## Author Contributions

Z-NC, HB, and J-JL designed the study. J-JL and HW performed the experiments and analyzed the data. J-JL wrote the manuscript. HB and J-JL revised the manuscript. H-YC, Z-KL, R-YZ, MLu, CL, Y-LY, MLi, HZ, T-JZ, KZ, GL, GN, CZ, S-PG, and LW provided experimental material and technical support. All authors approved the final manuscript.

## Conflict of Interest

The authors declare that the research was conducted in the absence of any commercial or financial relationships that could be construed as a potential conflict of interest.

## Supplementary Material

Supplementary Figure 1 **(A)** The levels of fasting lipid profiles of C57BL/6J mice or *ApoE^–/–^* mice after being fed a normal chow diet or a Western diet for 16 weeks. *n* = 6. Data represent the mean ± SEM. **(B)** CD147 IHC scoring. Analysis of contingency, Fisher’s exact test. ^∗∗∗^*P* < 0.001, ^****^*P* < 0.0001. **(C)** Immunohistochemical detections of CD147 in mouse aortic sinus. The scale bar is 200 μm. *n* = 6. **(D)** Western blot analysis of CD147 protein levels in BMDMs and pMacs exposed to 50 μg/mL ox-LDL for 24 h in the presence or absence of NF-κB. Data represent the mean ± SEM. of *n* = 3 biologically independent experiments. **(E)** GSEA enriched gene sets involved in atherosclerosis are illustrated as heatmaps and include LDL clearance, plasma lipoprotein clearance, platelet aggregation, and collagen degradation.

## Abbreviations

3-MA: 3-Methyladenine
ABC: ATP-binding cassette
ac-LDL: acetylated-low-density lipoprotein
BMDM: bone marrow-derived macrophage
DAPI: 4’,6-diamidino-2-phenylindole
DEG: differentially expressed gene
FDR: false discovery rate
GO: Gene Ontology
GSEA: Gene Set Enrichment Analysis
H&E: hematoxylin and eosin
KEGG: Kyoto Encyclopedia of Genes and Genomes
MMP: matrix metalloproteinase
NES: normalized enrichment score
ox-LDL: oxidative-low-density lipoprotein
PFA: paraformaldehyde
pMacs: peritoneal macrophages
RT-PCR: real-time PCR
SR-A: scavenger receptor-A.
