# Supplementary figures and images for: Blockade of Macrophage CD147 Protects Against Foam Cell Formation in Atherosclerosis
Source: Front Cell Dev Biol. 2021 Jan 8;8:609090. doi: 10.3389/fcell.2020.609090 (PMC7820343; doi:10.3389/fcell.2020.609090)

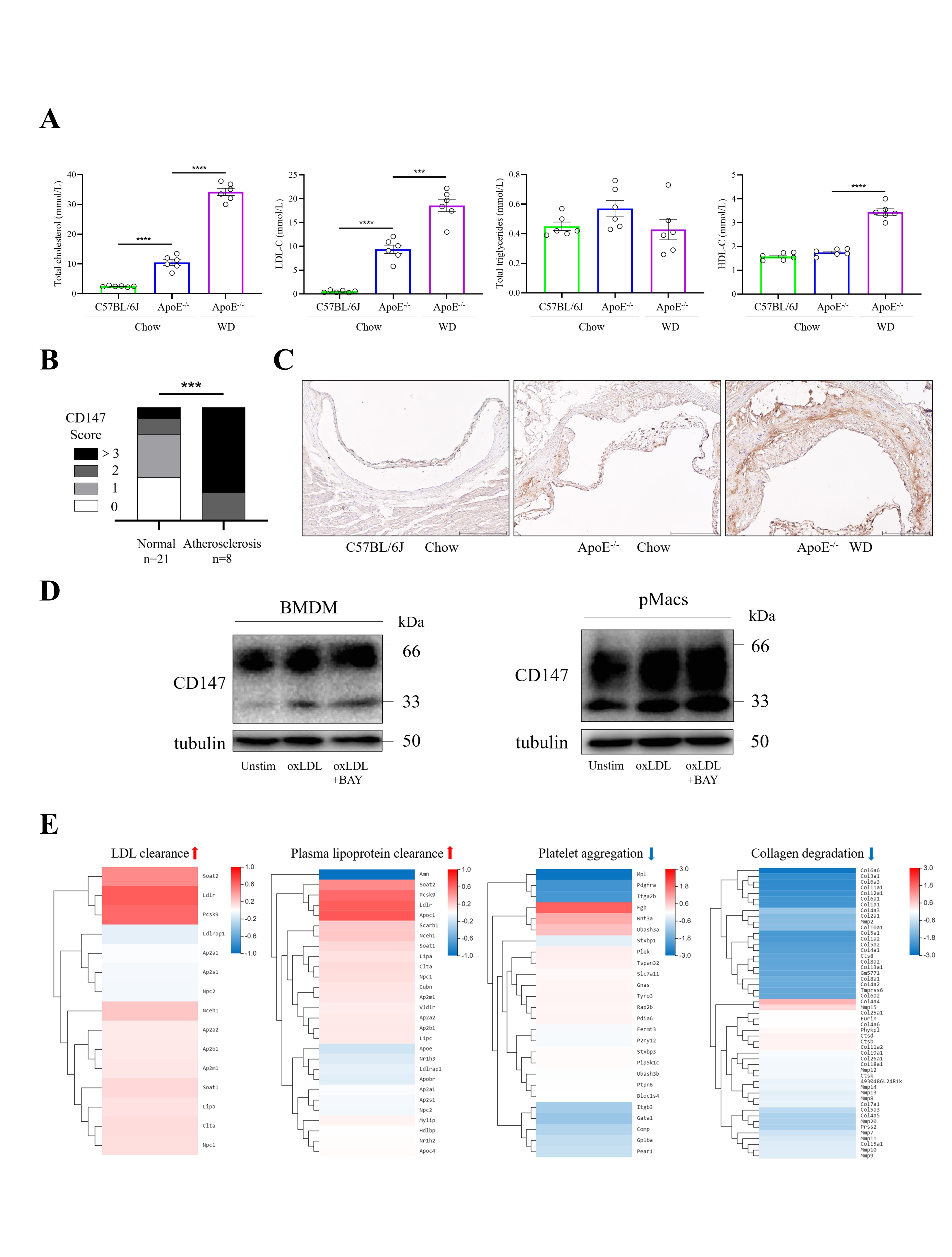

Supplement: Supplementary Figure 1 — (A) The levels of fasting lipid profiles of C57BL/6J mice or ApoE–/– mice after being fed a normal chow diet or a Western diet for 16 weeks. n = 6. Data represent the mean ± SEM. (B) CD147 IHC scoring. Analysis of contingency, Fisher’s exact test. ∗∗∗P < 0.001, ****P < 0.0001. (C) Immunohistochemical detections of CD147 in mouse aortic sinus. The scale bar is 200 μm. n = 6. (D) Western blot analysis of CD147 protein levels in BMDMs and pMacs exposed to 50 μg/mL ox-LDL for 24 h in the presence or absence of NF-κB. Data represent the mean ± SEM. of n = 3 biologically independent experiments. (E) GSEA enriched gene sets involved in atherosclerosis are illustrated as heatmaps and include LDL clearance, plasma lipoprotein clearance, platelet aggregation, and collagen degradation. [file Image_1.JPEG]
